# First report on molecular prevalence and identification of *Anaplasma platys* in dogs in Khon Kaen, Thailand

**DOI:** 10.14202/vetworld.2021.2613-2619

**Published:** 2021-10-06

**Authors:** Biethee Rani Sarker, Thongphet Mitpasa, Arayaporn Macotpet, Pattara-Anong Bupata, Somboon Sangmaneedet, Weerapol Taweenan

**Affiliations:** 1 Division of Pathobiology, Faculty of Veterinary Medicine, Khon Kaen University, Khon Kaen 40002, Thailand; 2 Division of Medicine, Faculty of Veterinary Medicine, Khon Kaen University, Khon Kaen 40002, Thailand; 3 Veterinary Teaching Hospital, Faculty of Veterinary Medicine, Khon Kaen University, Khon Kaen 40002, Thailand.

**Keywords:** *Anaplasma platys*, molecular prevalence, nested polymerase chain reaction, thrombocytotropic anaplasmosis

## Abstract

**Background and Aim:**

*Anaplasma platys* is a blood parasite that infects platelets, causing thrombocytopenia. *Rhipicephalus sanguineus* ticks are believed to transmit *A. platys*. To identify *A. platys*, nested polymerase chain reaction (PCR) has proven to be an effective diagnostic tool. In this study, the molecular prevalence of *A. platys* infection in dogs was investigated for the 1^st^ time in the Khon Kaen region of Thailand. The association between risk factors and *A. platys* infection was also evaluated.

**Materials and Methods:**

A total of 130 blood samples were collected from dogs in Khon Kaen, Thailand. DNA from the samples was extracted and nested PCR was applied for molecular analysis. Platelet count and packed cell volume (PCV) levels were measured. Platelet counts were categorized into four grades: Non-thrombocytopenia (platelets >200,000 cells/μL), mild thrombocytopenia (platelets 150,000-200,000 cells/μL), moderate thrombocytopenia (platelets 100,000-150,000 cells/μL), and severe thrombocytopenia (platelets <100,000 cells/μL). Four categories for PCV levels of >37%, 30-37%, 20-29%, and <20% were defined as no anemia, mild anemia, moderate anemia, and severe anemia, respectively. DNA sequencing was analyzed using BTSeq™ (Barcode-Tagged Sequencing; CELEMICS, Seoul, South Korea) for similarity index.

**Results:**

Among the 130 samples, 9 (6.9%) were positive for *A. platys* infection. There was an association between low platelet count and infection (p<0.05). PCV level was also associated with *A. platys* infection (p<0.05). DNA sequencing results of the nine positive samples showed similarity to known sequences of *A. platys* with 99.36-100% nucleotide identity. These results suggested low genetic diversity in *A. platys* infecting dogs in the Khon Kaen area.

**Conclusion:**

By amplifying *16S rRNA*, *A. platys* infection was detected in the blood of Thai dogs. Further work should be performed to identify risk factors potentially associated with *A. platys* infection in dogs in Khon Kaen. Other related factors should also be considered, such as location and breeding, as well as the environmental characteristics of each locality. In addition, sampling a larger number of animals may reveal predictors for the positivity of *A. platys* in dogs in this region.

## Introduction

Dog ownership is increasingly common and is known to improve both physical health and mental well-being in owners. However, dog ownership is associated with some potential risks, including the transmission of disease from pets to humans. According to the Bureau of Disease Control and Veterinary Sciences, there are around 8.5 million dogs in Thailand [1]. Different types of parasite can be carried by dogs, which can also present a health risk to humans [2]. Some parasites can be blood-borne, so they can be found in the bloodstream of infected animals. Many blood-borne parasites are spread by insects (vectors). *Anaplasma platys* is one of the most important parasites and causes major tick-borne disease in dogs of all ages. *A. platys* infects platelets and is the cause of thrombocytotropic anaplasmosis in canine species. Due to the cyclic nature of this disease, seeing the microorganism inside the platelets of a diseased animal’s blood is difficult and may be an incidental finding [3]. *Rhipicephalus sanguineus* ticks are believed to transmit *A. platys* because the DNA of *A. platys* has frequently been found in these ticks worldwide. South Europe is known as an endemic region for *A. platys* and there are reports of this infection in Greece [4], Albania [5], Portugal [6], Spain [7], France [8], Romania [9], and Italy [10]. Despite diversity between two *Anaplasma* spp., commercial ELISA kits are reliable for diagnosing *A. platys* infection serologically because they utilize a cross-reaction of antibodies against *Anaplasma phagocytophilum* and *A. platys* [11]. Clinical signs including fever, lethargy, anorexia, pale mucous membranes, petechiae, and lymphadenomegaly are commonly seen in *A. platys* infection. For the diagnosis of *A. platys*, polymerase chain reaction (PCR) is commonly used due to its high specificity and sensitivity. Nested PCR is 10 times more sensitive than single PCR. A previous study confirmed the usefulness of nested PCR for assessing the duration of antibiotic treatment required for dogs infected with *A. platys* [12].

To the best of our knowledge, *A. platys* infection in dogs has not yet been investigated in the Khon Kaen region, Thailand. Nevertheless, Maha Sarakham and Kalasin Provinces (the nearest Thai Provinces to Khon Kaen) have been reported as areas endemic for the tick vector *R. sanguineus* and also for *A. platys* infection [13].

Against this background, the aims of the current study were to investigate the molecular prevalence of *A. platys* from blood samples of dogs in Khon Kaen Province and to evaluate the association between risk factors and *A. platys* infection. This study will help to understand the prevalence of the infection in Khon Kaen and can contribute to improving the control and prevention of *A. platys* infection.

## Materials and Methods

### Ethical approval

The use of animals in the current study was approved under permissions and the guidelines of the Institutional Animal Care and Use Committee of Khon Kaen University (KKU) as permission record no. IACUC-KKU-8/64 and reference no. 660201.2.11/45 (22).

### Study period, area, and population

**Figure-1:**
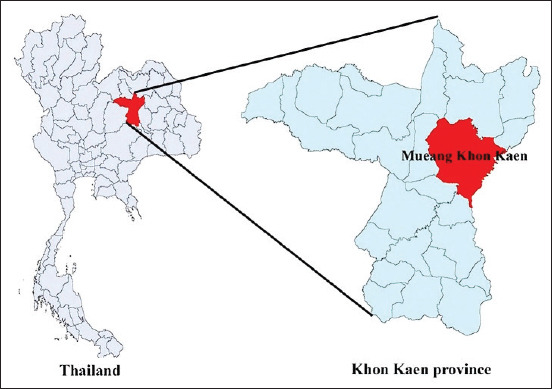
Location of the study area (Source: https://doi.org/10.1186/s12889-018-5871-1).

During May-November 2020, blood samples were collected from dogs visiting private animal clinics in Mueang district and KKU Veterinary Teaching Hospital, Khon Kaen, Thailand (Figure-1). This was a cross-sectional study. A total of 130 blood samples were collected from the cephalic vein or saphenous vein of domestic dogs. The sample size calculation was based on the reported prevalence of *A. platys* in domestic dogs in Thailand of 29.4% [14], with 10% error allowable. The samples were collected randomly from dogs coming to the KKU Veterinary Teaching Hospital and private animal clinics with the owners’ consent. There was no age or sex limitation for inclusion in this study. Blood samples were collected in blood collecting tubes with EDTA, stored in an icebox, and finally brought to the Faculty of Veterinary Medicine, KKU. Each blood sample was divided into two aliquots for hematological and molecular detection. The hematological analysis evaluated the platelet count and packed cell volume (PCV).

### Evaluation of platelet count

An aliquot of a 0.5 mL blood sample was sent to the Vet Central Lab, Khon Kaen, for hematological analysis. Platelet counts were categorized into four grades: severe thrombocytopenia (platelets <100,000 cells/µL), moderate thrombocytopenia (platelets 100,000-150,000 cells/µL), mild thrombocytopenia (platelets 150,000-200,000 cells/µL), and non-thrombocytopenia (platelets >200,000 cells/µL) [14].

### Evaluation of PCV level of blood

The PCV level of blood was also measured to understand the relationship between anemia and *A. platys* infection. First, two-thirds of a heparinized microhematocrit tube or capillary tube was filled with blood. Then, the tube was placed into a calibrated microhematocrit centrifuge machine. After that, the height of the red cell layer was measured by a hematocrit reader. For interpretation of the PCV of blood, the four categories >37%, 30-37%, 20-29%, and <20% were classified as no anemia, mild anemia, moderate anemia, and severe anemia, respectively [14]. These PCV and platelet counts were measured at the Vet Central Lab, Khon Kaen, Thailand.

### DNA extraction

DNA was extracted from 200 μL of each anticoagulated blood sample using a GF-1 blood DNA extraction kit (Vivantis, Malaysia), in accordance with the manufacturer’s instructions.

### Nested PCR assay

The *16s rRN*A gene of *A. platys* was amplified by nested PCR using universal primers for *Rickettsia* (ECC and ECB) and *A. platys*-specific primers (PLATYS and GA1UR) in the first and second steps, respectively [13] (Table-1).

**Table-1:** Universal *Rickettsia* and *Anaplasma platys-*specific primers and corresponding product size.

Primer	Sequence	Product size (bp)
ECC (F)	5’AGA-ACG-AAC-GCT-GGC-GGC-AAG-CC 3’	478
ECB (R)	5’ CGT-ATT-ACC-GCG-GCT-GCT-GGC-A 3’	
PLATYS (F) GA1UR (R)	5’ TTT-GTC-GTA-GCT-TGC-TAT-G 3’	402
	5’ GAG-TTT-GCC-GGG-ACT-TCT-TCT 3’	

The PCR reaction consisted of approximately 50 ng of extracted DNA, 10 pmol each primer, 200 μM each dNTP, 1.5 mM MgCl_2_, and 1 unit Taq polymerase (Vivantis). PCR conditions were initial denaturation at 95ºC for 2 min; 35 cycles of denaturation at 95°C for 1 min, annealing at 60°C and 62°C for the 1^st^ and 2^nd^ steps for 1 min, and extension at 72°C for 2 min; and then final extension at 72°C for 5 min in a Biometra GmbH Thermocycler (Germany). After that, PCR products were identified in 1% agarose gels and visualized under ultraviolet light [13].

### DNA sequencing and analysis of obtained sequences

After purification with a gel purification kit (Vivantis), all positive samples were sent for sequencing using BTSeq™ (Barcode-Tagged Sequencing; CELEMICS, Seoul, South Korea). The sequences were compared to other *A. platys* reference sequences in GenBank using BLAST search. MEGA X software (Molecular Evolutionary Genetics Analysis software available at https://www.megasoftware.net/) was used to align samples with the GenBank database for confirmation of the status as *A. platys* and to construct a phylogenetic tree based on the neighbor-joining method with 500 replicates for bootstrap analysis.

### Statistical analysis

Risk factors, including PCV and platelet count were evaluated for the association with the molecular prevalence using a logistic regression test in the IBM SPSS Statistics program, Version 17 (IBM, NY, USA). The prevalence (%), p-value, and 95% confidence interval were calculated. p<0.05 was considered statistically significant.

## Results

**Figure-2:**
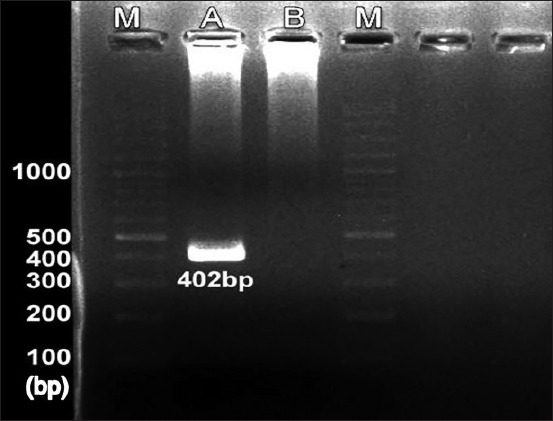
After gel electrophoresis, samples show the positive band with 402 bp. Lane M: Ladder, lane A: Positive sample, lane B: Negative control.

A total of 130 blood samples from dogs were collected randomly from seven different private animal clinics and KKU Veterinary Teaching Hospital, Khon Kaen, Thailand. Among these 130 samples, 9 (6.9%) were found to be positive by PCR. Of these nine positive samples, eight were from the KKU Veterinary Teaching Hospital and one from a private clinic. All nine samples showed positivity with a clear band size (402 bp) (Figure-2).

The associations between the prevalence of *A. platys* infection and risk factors including rearing status and tick infestation were evaluated. The results showed that nine samples were found positive for *A. platys*. According to the tick infestation factor, the prevalence of the dogs with the presence of tick was 11.8% (8/68), whereas the prevalence of the dogs with no tick found was 1.6% (1/62) (Table-2). Moreover, the infection rates in dogs confined to the house, allowed into the yard, and free-roaming were 5.9% (1/17), 4.2% (3/72), and 12.2% (5/41), respectively (Table-2). The results showed that rearing status and tick infestation were not individual factors significantly associated with *A. platys* infection, but in combination, an association between tick infestation and infection was identified (Table-2).

**Table-2:** Prevalence of *Anaplasma platys* infection and some related statistical characteristics (risk factors).

Variables	% prevalence (no. of positive/no. of samples)	Univariable analysis		Multivariable analysis	
	
		OR (95% CI)	p*-*value	Adjusted OR (95% CI)	p*-*value
Tick infestation					
No tick found	1.6 (1/62)	1		1	
Presence of tick	11.8 (8/68)	8.13 (0.99-67.03)	0.05	15.25 (1.23-187.84)	0.03
Rearing status					
Around the house	4.2 (3/72)	1		1	
Raise in room	5.9 (1/17)	1.43 (0.14-14.74)	0.76	0.25 (0.02-3.19)	0.29
Independent	12.2 (5/41)	3.19 (0.72-14.13)	0.13	0.56 (0.09-3.39)	0.52

### Measurement of platelet count and PCV value

According to platelet counts, the prevalence in dogs with severe thrombocytopenia, moderate thrombocytopenia, mild thrombocytopenia, and non-thrombocytopenia group was 15.8% (3/19), 26.7% (4/15), 7.7% (1/13), and 1.2% (1/83), respectively (Table-3). Most of the dogs (83/130; 63.8%) were in the non-thrombocytopenia group. Platelet count was significantly associated with *A. platys* infection in this study (p=0.04, Table-3). The table shows that the moderate thrombocytopenia group was most commonly observed in infected dogs (26.7%; 4/15) (Table-3).

**Table-3:** Factors associated with platelet count and PCV level in reference to *Anaplasma platys* infection.

Variable	% prevalence (no. of positive/no. of samples)	Univariable analysis		Multivariable analysis	
	
		OR (95% CI)	p*-*value	Adjusted OR (95% CI)	p-value
Platelet count			0.04		0.46
Severe thrombocytopenia <100,000 Cell/mL	15.8 (3/19)	20.50 (1.97-213.42)	0.007	3.17 (0.05-216.12)	0.59
Moderate thrombocytopenia (100,000-150,000) cell/mL	26.7 (4/15)	21.87 (2.28-209.42)	0.012	8.74 (0.11-683.41)	0.33
Mild thrombocytopenia (150,000-200,000) cell/mL	7.7 (1/13)	6.83 (0.4-116.63)	0.184	1.74 (0.02-123.69)	0.8
Non-thrombocytopenia >200,000 cell/mL	1.2 (1/83)	1			
PCV level			0.024		0.26
Severe anemia (<20%)	28.6 (4/14)	34.40 (3.49-338.70)	0.002	16.36 (0.26-1020.67)	0.19
Moderate anemia (20-29%)	18.2 (2/11)	19.11 (1.57-232.06)	0.021	5.57 (0.06-496.39)	0.45
Mild anemia (30-37%)	11.1 (2/18)	10.75 (0.92-125.71)	0.058	2.08 (0.03-165.05)	0.74
No anemia (>37%)	1.1 (1/87)	1			

The PCV values were categorized into four levels. PCV <20%, 20-29%, 30-37%, and >37% were categorized as severe anemia, moderate anemia, mild anemia and no anemia, respectively. The results indicate that the prevalence of *A. platys* infection in dogs with severe anemia, moderate anemia, mild anemia, and no anemia was 28.6% (4/14), 18.2% (2/11), 11.1 (2/18), and 1.1 (1/87), respectively (Table-3). Most of the dogs (87/130; 67%) were in the no anemia group. The severe anemia group was associated with a higher risk of infection than the mild, moderate, and no anemia groups. The association between PCV level and *A. platys* infection was significant (p= 0.024) in this study. Combined effects of platelet count and PCV level showed no association with *A. platys* infection (Table-3).

### Phylogenetic relationship of revealed *A. platys*-like blood parasite with known strains

**Figure-3:**
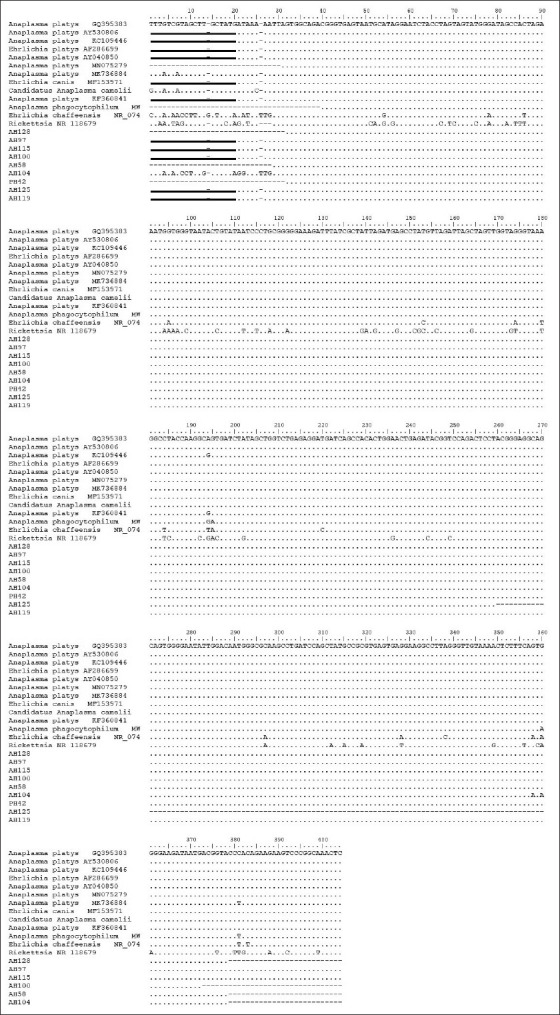
Alignment of the sequences obtained with the *16S rRNA* used in our study and those of *A. platys* in GenBank. (..)denotes the identical nucleotide sequence to that of *A. platys*. (--) denotes absence of nucleotides and (__) denotes primer sequence.

To evaluate phylogenetic relationships, the sequences of *A. platys* variants in this study and those described previously were compared to each other. These nine gene sequences shared 99.36-100% nucleotide identity with known sequences of *A. platys*. For example, PH42 and AH52 shared 100% nucleotide identity with *A. platys* strain S3 chromosome CP046391. Meanwhile, AH97 showed 99.78%, and AH119, AH100, and AH128 showed 99.78%, 99.76%, and 99.75% identity to *A. platys* strain S3 chromosome CP046391, respectively. Moreover, AH125 and AH115 showed 99.67% and 99.36% similarity to *A. platys* isolate D35 ribosomal RNA KX792089. Alignment of the sequences from the current study and those of reference *A. platys* in GenBank indicated identical nucleotide sequences of *A. platys* (Figure-3). The alignment showed that there were differences in some bases in the outgroups *Ehrlichia canis* MF153971, *Candidatus Anaplasma* MN882724, *Ehrlichia chaffeensis* NR074500, and *Rickettsia* NR 118679. *A. platys* MK736884 and sample AH104 also showed differences in the aligned part.

**Figure-4:**
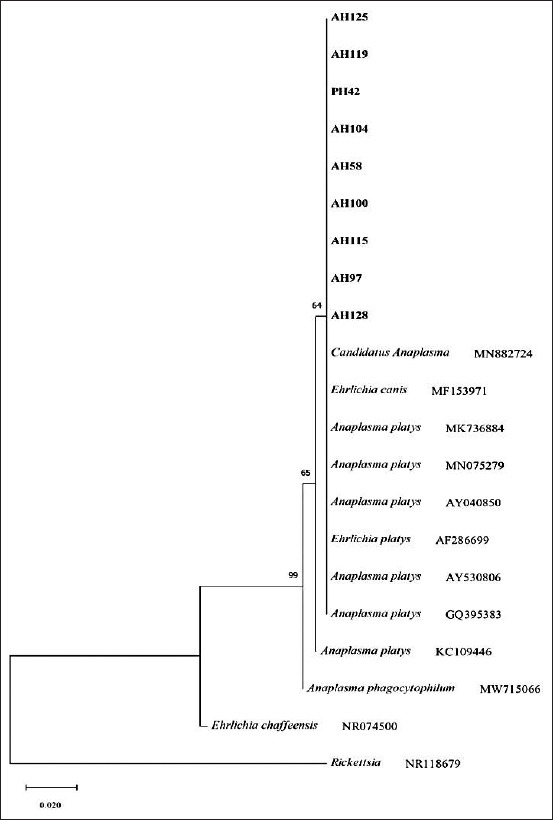
Phylogenetic tree based on 16S RNA gene and drawn using MEGA X software. Trees were obtained by neighbor-joining method. The numbers at the nodes are the proportions of 500 bootstrap with Kimura 2-parameter model. Samples detected in this work were highlighted.

The phylogenetic tree showed that the samples PH42, AH58, AH97, AH100, AH104, AH115, AH119, AH125, and AH128 were closely related to each other and matched with references *A. platys* AY040850, *A. platys* GQ395383, *A. platys* AY530806, *Ehrlichia platys* AF286699, *A. platys* MN075279, and *A. platys* MK736884 (Figure-4). According to the phylogenetic tree, the outgroups *E. canis* MF153971 and *Candidatus Anaplasma* MN882724 showed similarity with *A. platys* sequences, but *E. chaffeensis* NR074500, *A. phagocytophilum* MW715066, and *Rickettsia* NR 118679 did not.

## Discussion

This study molecularly investigated the prevalence of *A. platys* infection in dogs in Khon Kaen Province, Thailand. Samples from KKU Veterinary Teaching Hospital covered dogs from a large area of Khon Kaen, whereas private clinics covered smaller populations. In this study, 6.9% of the PCR results were positive, which is a different rate of *A. platys* infection as identified by PCR evaluation in other provinces of Thailand. Previously, *A. platys* infections were reported in multiple studies in different provinces in Thailand, for example, Kalasin Province (29.4%) [14], Maha Sarakham Province (29.2%) [13], and Buriram Province (30.61%) [15], but at different rates from the current study.

However, the prevalence in the present study is similar to a result reported in Songkhla (4.4%), where samples were collected from stray dogs [16], although the present study samples were collected only from hospitalized dogs. Location and climate are factors potentially influencing this variation of results. In Brazil, a study revealed a high prevalence of *A. platys* infection of 32.9% [17]. Climatic variation, the dog rearing system, and the prevalence of ticks, among others, may be behind these differences.

An association was found between PCV level and infection (p=0.024), which contrasts with the findings in studies from Kalasin (p=0.294) and Maha Sarakham Provinces (p=0.816). Regarding platelet count, a significant association between platelet count and *A. platys* infection was identified (p=0.04). This conclusion differs from findings in the previous studies in Thailand at Kalasin (0.807%) and Maha Sarakham Provinces. Sample collection strategy could be a factor behind this discrepancy, with the present study only focusing on hospitalized dogs.

The close relationship between 16S rDNA of *A. platys* from various regions worldwide agrees with the hypothesis that *A. platys* strains are not separated geographically [18]. Figure-4 displays that the phylogenetic tree was separated into four major clusters and *A. platys* Khon Kaen was within the same cluster. Within these four clusters, little genetic diversity was observed, suggesting slow and homogeneous evolution [19]. In this study, *A. platys* was also found to be more closely related to *E*. *platys*, which supports an earlier study [20].

As suggested in the present work, the few differences in the *16S rRNA* gene sequences compared to those available in GenBank might be due to sequencing or PCR error, but may be due to variations in the sequences of strains of the same species as well. Finally, our primers detected *A. platys* in canine blood, which resulted in new *16S rRNA* sequences being obtained from *A. platys* infections of Thai dogs. Although the *16S rRNA* sequences were highly conserved among geographically diverse strains of these organisms, additional analyses of genes were recommended (e.g., outer membrane protein gene families), which could help elucidate the diversity and evolution of strains from different geographical areas.

## Conclusion

The nested PCR method employed “two-step” direct amplification using an *A. platys*-specific primer set. To increase the sensitivity of the PCR, a nested PCR approach was developed in this study. This study provides epidemiological information about the prevalence of pathogens transmitted by ticks in Khon Kaen Province to establish control and preventive measures against *A. platys* infection in dogs. Although light microscopy analysis appeared to be a reliable method to diagnose *A. platys* infection, the use of PCR was more specific to identify this parasite. The results show that these pathogens circulate among canines in the Khon Kaen region of Thailand. The results of a phylogenetic analysis demonstrated very high homology values (100%) with a bootstrap value of 100%. This also shows that there is low genetic diversity in *A. platys* infecting dogs in the Khon Kaen area. The identification of risk factors that may be associated with *A. platys* infection in dogs in Khon Kaen should be further investigated, such as factors related to the location and breeding, as well as the environmental characteristics of each locality. In addition, sampling a larger number of animals may demonstrate the possible risk factors influencing the positivity of dogs for *A. platys* in this region. The development of molecular techniques appears to facilitate the identification of these diseases, and information on the occurrence of ticks and pathogens transmitted by them will help veterinary services to diagnose diseased animals correctly.

## Authors’ Contributions

WT: Conceptualization, funding acquisition, project administration, and reviewed and revised the manuscript. BRS and WT: Data curation. methodology, and visualization. BRS: Formal analysis, software, and drafted the manuscript. TM, BRS, AM, and PB: Investigation. TM, AM, and PB: Resources. SS: Supervision. WT and SS: Validation. All authors read and approved the final manuscript.
